# Lateral femoral cutaneous nerve transposition

**DOI:** 10.3171/2022.10.FOCVID2289

**Published:** 2023-01-01

**Authors:** Garret P. Greeneway, Paul S. Page, Simon G. Ammanuel, Amgad S. Hanna

**Affiliations:** Department of Neurological Surgery, University of Wisconsin School of Medicine and Public Health, Madison, Wisconsin

**Keywords:** entrapment neuropathy, lateral femoral cutaneous nerve, meralgia paresthetica, nerve transposition

## Abstract

Lateral femoral cutaneous neuropathy, also known as meralgia paresthetica, is a pathology commonly encountered by neurosurgeons. Symptoms include numbness, tingling, and burning pain over the anterolateral thigh due to impingement on the lateral femoral cutaneous nerve (LFCN). Surgical treatment has traditionally involved nerve release or neurectomy. LFCN transposition is a relatively new approach that can provide excellent symptomatic relief. In this video, the authors highlight key operative techniques to ensure easy identification, adequate decompression, and transposition of the nerve. Key steps include ultrasound-guided wire localization, superficial decompression, opening of the inguinal ligament, deep decompression, and medial transposition.

The video can be found here: https://stream.cadmore.media/r10.3171/2022.10.FOCVID2289

## Transcript

### 0:27

Patients with lateral femoral cutaneous neuropathy, also known as meralgia paresthetica, typically present with numbness, tingling, and burning pain over the anterolateral thigh due to impingement of the lateral femoral cutaneous nerve (LFCN). Surgery is often offered to patients when symptoms are intractable and when conservative measures fail. A simple surgical decompression is associated with a high failure rate.^1^ Neurectomy is associated with sensory loss and can cause deafferentation pain. Until recently, there have been very few reports of transposing the LFCN in the literature.^2,3^

### 1:07

Due to unsatisfactory outcomes with simple decompression and neurectomy, and an unclear direction for surgically managing meralgia paresthetica, a cadaveric study was published to aid in developing a better understanding of the anatomical intricacies of the LFCN canal.^4^ This study led to the publishing of an anatomical feasibility study that describes deep decompression of the nerve and a medial transposition technique.^5^ Here is a diagrammatic representation of the LFCN canal in an oblique sagittal view. After simple decompression, the fascia superficial to the nerve is released, as is the inguinal ligament. When scar tissue develops, the nerve is retethered into a position very similar to that in the preoperative state. With transposition, all components of the LFCN canal are opened superficial and deep to the nerve, and then the nerve is mobilized approximately 2 cm medially. After transposition, the nerve acquires a much straighter and relaxed course, has a softer muscular bed, and is further away from the anterior superior iliac spine (ASIS), even after scar tissue develops.

### 2:12

The literature now suggests that deep decompression and transposition of the LFCN provide better results than simple decompression alone.^6^ Here we present a surgical demonstration of a left LFCN transposition in a patient with refractory meralgia paresthetica that has failed conservative management.

### 2:34

The patient is placed in the supine position on a stretcher in the radiology suite for ultrasound-guided wire localization of the LFCN.^7^ Clear adhesive skin tape is used to hold the patient’s rostrally mobilized abdominal fat pannus in place, allowing for more direct access to the nerve.

### 2:58

A diagnostic ultrasound (US) is first performed to locate the nerve. The nerve (blue arrow) and its canal (yellow arrow) are readily visible on the US image. The skin overlying the left LFCN is marked, sterilized, and draped. Superficial and deep soft tissues are anesthetized with a buffered 1% lidocaine local anesthetic. Utilizing US, a 5-cm Homer wire localization needle is advanced at a 45° angle just deep to the left LFCN prior to deploying the wire with the hook coursing around the nerve. A static ultrasound image showing the nerve to skin surface and needle to skin surface is then obtained. The needle is then draped with sterile gauze and Tegaderm adhesive, and the patient is then transferred to the operating room theater.

### 4:09

The patient is positioned supine on a standard operating room table. Two-inch silk tape is used to mobilize the patient’s fat pannus rostrally out of the surgical field. Based on radiology measurements, a skin mark is made with a surgical marking pen estimating the location of the nerve. The anterior superior iliac spine is then palpated and marked. Palpation is then utilized to locate and mark the pubic tubercle. A dotted line is marked from the pubic tubercle to the anterior superior iliac spine, hereby estimating the approximate location of the inguinal ligament. A linear incision is then planned and marked using the distance as measured in the radiology suite between the entry point of the protruding needle and the perceived cross-sectional plane of the nerve. The incision is approximately 5 to 6 cm in length and planned along the course of the nerve just beneath the inguinal ligament. The surgical field is then prepped, draped, and local anesthetic is infiltrated subcutaneously, taking care to avoid injection into the nerve.

### 5:43

The skin and dermis are sharply opened using a 15 blade scalpel, exposing subcutaneous fat. Dissection through subcutaneous fat is performed using a combination of Metzenbaum scissors and Bovie monopolar electrocautery until the LFCN canal is encountered. Self-retaining retractors are placed and Army-Navy retractors are utilized to aid in visualization. The wire is then observed traversing the sartorius fascia at an approximately 45° angle.

### 6:24

Superficial decompression is achieved by meticulously opening the superficial layer of the LFCN canal, based off of wire localization, using a Metzenbaum scissors. Yellow vessel loops are placed around the nerve in order to identify and protect it during the decompression. The decompression is carried distally and proximally until the inguinal ligament is encountered.

### 7:00

The inguinal ligament is then probed and inspected to ensure there are no neurovascular structures in the immediate inferior aspect of the ligament. Using a Metzenbaum scissors, the inguinal ligament is carefully opened. Attention is then directed toward the floor of the LFCN canal for the deep decompression portion of the procedure.

### 7:24

For the deep decompression portion of the procedure, a deep, white, and glistening fascial layer is noted deep to the path of the nerve. Here is an image captured from a cadaveric demonstration. The white arrow identifies the deep, white, and glistening fascial layer deep to the path of the nerve. The deep fascial layer is opened using a Metzenbaum scissors. Opening of this fascial layer, as well as the septum medial to sartorius muscle, allows for the nerve to be medially transposed away from the anterior superior iliac spine.

### 7:58

The medial transposition portion of the procedure ensues following the superficial decompression, deep decompression, and inguinal ligament release. The nerve is now decompressed in a 360° fashion and ready for medial transposition. Medially transposing the nerve away from the anterior superior iliac spine hereby allows for the nerve to have a shortened and much relaxed course. A small pocket is then created in the soft tissues just medial to the LFCN canal to harbor the medially transposed nerve. A 3-0 Vicryl suture can be placed to ensure that the nerve remains medially transposed in the newly created tissue pocket. The nerve is then carefully inspected to ensure that it is circumferentially decompressed, under no tension, and freely transposed. We are typically able to transpose the nerve medially by approximately 2 cm, as pictured here.

### 9:05

Hemostasis is obtained with bipolar electrocautery, and the wound is then copiously irrigated with antibiotic-impregnated saline. A small stab incision is made adjacent to the surgical site using a 15 blade, and a 7-Fr round J-P drain is gently tunneled into the surgical site. The drain is secured in place with a 3-0 nylon purse string stitch and then connected to a Jackson-Pratt silicone bulb. Attention is then diverted to closure of the wound in a layered fashion. First, the subcutaneous fat layer and dermal layers are both closed using 3-0 Vicryl sutures placed in an inverted fashion to bury the knot. The skin is then closed in a simple running fashion using a 3-0 nylon suture. The incision is then gently cleaned with wet and dry surgical sponges, and a silver Mepilex dressing is applied to cover the incision. No immobilization is required, and the patient is allowed to bear weight immediately.

## Disclosures

The authors report no conflict of interest concerning the materials or methods used in this study or the findings specified in this publication.

## Author Contributions

Primary surgeon: Hanna. Editing and drafting the video and abstract: all authors. Critically revising the work: all authors. Reviewed submitted version of the work: all authors. Approved the final version of the work on behalf of all authors: Hanna. Supervision: Hanna.

## Supplemental Information

### Patient Informed Consent

The necessary patient informed consent was obtained in this study.
